# Response of pest control by generalist predators to local‐scale plant diversity: a meta‐analysis

**DOI:** 10.1002/ece3.1917

**Published:** 2016-01-25

**Authors:** Anicet Gbèblonoudo Dassou, Philippe Tixier

**Affiliations:** ^1^ CIRADPersystUPR 26TA B‐26/PS4, Boulevard de la Lironde34398Montpellier Cedex 5France; ^2^ CARBAPAfrican Research Centre on Bananas and PlantainsBP 832DoualaCameroon; ^3^ Laboratory of Biotechnology, Genetic Resources and Plant and Animal Breeding (BIORAVE)Faculty of Sciences and Technology of DassaPolytechnic University of Abomey01 BP 14Dassa‐ZoumèBenin; ^4^ Departamento de Agricultura y AgroforesteriaCATIE7170 CartagoTurrialba30501Costa Rica

**Keywords:** Biological control, effect size, herbivores, local scale, pest regulation service, plant diversity, predation, specialization

## Abstract

Disentangling the effects of plant diversity on the control of herbivores is important for understanding agricultural sustainability. Recent studies have investigated the relationships between plant diversity and arthropod communities at the landscape scale, but few have done so at the local scale. We conducted a meta‐analysis of 32 papers containing 175 independent measures of the relationship between plant diversity and arthropod communities. We found that generalist predators had a strong positive response to plant diversity, that is, their abundance increased as plant diversity increased. Herbivores, in contrast, had an overall weak and negative response to plant diversity. However, specialist and generalist herbivores differed in their response to plant diversity, that is, the response was negative for specialists and not significant for generalists. While the effects of scale remain unclear, the response to plant diversity tended to increase for specialist herbivores, but decrease for generalist herbivores as the scale increased. There was no clear effect of scale on the response of generalist predators to plant diversity. Our results suggest that the response of herbivores to plant diversity at the local scale is a balance between habitat and trophic effects that vary according to arthropod specialization and habitat type. *Synthesis and applications*. Positive effects of plant diversity on generalist predators confirm that, at the local scale, plant diversification of agroecosystems is a credible and promising option for increasing pest regulation. Results from our meta‐analysis suggest that natural control in plant‐diversified systems is more likely to occur for specialist than for generalist herbivores. In terms of pest management, our results indicate that small‐scale plant diversification (via the planting of cover crops or intercrops and reduced weed management) is likely to increase the control of specialist herbivores by generalist predators.

## Introduction

Pest regulation services in agriculture depend on a complex suite of direct and indirect interactions involving multiple herbivores and predators (Stowe et al. 2000; Vandermeer et al. 2010; Cardinale et al. 2011). The services provided differ depending on the nature of the arthropod and plant communities (Duffy 2002). Agroecologists have suspected that increases in plant diversity within agricultural fields change food web structure and thereby reduce herbivore abundance and crop damage (Pimentel 1961; Andow 1991; Letourneau et al. 2011). In more diversified agroecosystems, however, predators may feed on more abundant alternative prey, thus decreasing the control of pests (Holt 1977). Determining the effects of plant diversification on pest control and determining the scale at which plant diversity affects pest control remain key challenges for sustainable agriculture (Bianchi et al. 2006).

A number of recent studies have investigated the effects of plant diversity at the landscape scale (Tscharntke and Brandl 2004; Fahrig et al. 2011). In their meta‐analysis, Chaplin‐Kramer et al. (2011) demonstrated that the effect of landscape complexity (that we can assume to be correlated with plant and crop diversity at these scales, i.e., diversity indices applied to percentage of crop and natural areas) on crop pests and natural enemies depends on the type of organisms (including their status as generalists or specialists). We hypothesize that the relationship between scale and the effect of plant diversity on crop pests may depend on the life‐history traits (and especially dispersal traits) of both the pests and their natural enemies and that the local scale may be especially relevant for those organisms with intermediate to low dispersal abilities. Except in the specific case of crop rotation (Rusch et al. 2013), the management of plant diversity is more difficult at the landscape scale than at the field scale, that is, farmers can directly manage communities at the field or local scale, but not at the landscape scale. A recent biodiversity experiment demonstrated that changes in plant diversity greatly affected all communities at the local scale (Scherber et al. 2010). In their meta‐analysis based on articles published between 1998 and 2008, Letourneau et al. (2011) showed that plant diversification increases herbivore suppression and the abundance of natural enemies and decreases crop damage. In their analysis, however, they did not separate specialist versus generalist predators, and they did not consider scale as a factor.

The effect of plant diversity on generalist predators remains a key topic in biological control (Moran and Hurd 1997; Altieri 1999; Scheu 2001; Ratnadass et al. 2012). Plant diversity may strongly influence omnivores, and the level of intraguild predation may increase in more diverse ecosystems (Rosenheim et al. 1993, 1995). Other mechanisms may counterbalance this potentially negative effect on pest regulation, that is, intraguild predation may be reduced in more structured and complex plant communities (Finke and Denno 2006). Theories based on modeling tend to show that increases in omnivory reduce the biological control of crop pests (Polis 1991; Diehl and Feißel 2000). This finding, however, has been challenged by recent studies. For instance, plant diversity experiments showed that abundance of all trophic groups except pests and invasive species increases with plant diversity (Scherber et al. 2010) and that the importance of predators increases as plant richness increases (Haddad et al. 2009).

The scale at which plant diversity is organized strongly influences its effects on communities (Thies et al. 2003; Stoner and Joern 2004) and more specifically on pest control (Bianchi et al. 2006). Results from Chaplin‐Kramer et al. (2011) suggest that the effects of plant diversification on the abundance of some predators may be stronger at the local scale than at larger scales. The importance of local scale was recently confirmed for natural enemies of arthropods (Sarthou et al. 2014). Some natural enemies are more responsive to factors at local than at larger scales (Thies et al. 2005; Perović et al. 2010). The appropriate scale for managing pest control and thus plant diversification is most likely linked to the dispersal capacities of both the pests and their predators. Although pest regulation involves multiscale processes (Thies and Tscharntke 1999; Schmidt et al. 2005; Rusch et al. 2010), it probably results principally from local processes when the predation occurs in a cropped field and when either the pest or its predators have limited dispersal capacities (Schellhorn et al. 2014). Furthermore, the reduced level of predation often observed in the center of fields (compared with field borders) clearly demonstrates how the lack of favorable habitat structure at the local scale may reduce pest control (Holzschuh et al. 2010). While landscape characteristics often determine the sources of pests or predators, local factors affect their final interactions at the field scale (Tylianakis and Romo 2010).

In this article, we present a meta‐analysis of the effects of plant diversity at the field scale on the control of pests by generalist predators. We used the meta‐analysis of 32 articles published between 2001 and 2014 (mostly after 2010) to determine how herbivore and predator specialization affects herbivore and predator responses to plant diversity and how these responses are affected by local scales ranging from 0.36 to 3300 m². Specially, we attempted to answer the following questions: (1) How does plant diversity affect herbivore and predator abundance and diversity? (2) Do generalist predators, specialist herbivores, and generalist herbivores respond differently to plant diversity? (3) Do generalist predators, specialist herbivores, and generalist herbivores respond differently to plant diversity at different local scales? We discuss the practical implications of our findings with respect to plant diversification and pest control in agricultural fields.

## Materials and Methods

### Study selection

We selected studies through a search on the Web of Science (last updated in June 2014) using the search string: [“plant diversity” OR “plant richness” OR “inter*crop*” OR “intercrop*”] AND [“predat*” OR “biological control” OR “pest control” OR “natural en*” OR “pest”] AND [“agr*” OR “crop”]. Over 559 abstracts were reviewed for relevance, and 32 papers were ultimately selected using the following criteria: (1) the study concerned plant diversity or intercropping, (2) the agroecosystem scale of the study was local (metric to field scale), and (3) statistics were reported regarding the relationship between plant diversity and arthropod response. The magnitude of plant diversity in these studies ranged between 1 and 96 species, with a mean value of 7.64 species (Table 1).

**Table 1:** List of 32 papers included in the meta‐analysis. The values are the number of each predicted variable (abundance, diversity, and plant damage) studied for predators and herbivores in the papers

Authors of papers	Predator		Herbivore		Plant Damage	Scale
	Abundance	Diversity	Abundance	Diversity		Surface area (m^2^)
Bickerton and Hamilton (2012)	3		2			19
Brose (2003)		1				1460
Cruz et al. (2013)	1	1				9
Diehl et al. (2013)		1		2		25
Fabian et al. (2014)	1		1			54
Fernandes et al. (2013)			2			54.6
Gámez‐Virués et al. (2010)	1		1			25
Haddad et al. (2009)	1	1	3	1		169
Haddad et al. (2011)	1	1	1	1		169
HansPetersen et al. (2010)	14		28			57.6
Hummel et al. (2012)	3	1				110
Lin et al. (2003)	2		1			200
Lin et al. (2011)			2			2400
Nakahira et al. (2012)	1		2			640
Nemec et al. (2014)	2					3025
Noman et al. (2013)	1		1			4
Nyasani et al. (2012)	4		18			50
Pitan and Odebiyi (2001)			4		1	72.6
Ramalho et al. (2012)	1		3			54.6
Sobek et al. (2009)	1		1			2500
Song et al. (2013)	5		1			720
Srinivasa Rao et al. (2012)	3		4			1000
Staudacher et al. (2013)			1		1	15
Stenchly et al. (2012)	1	1				1600
Straub et al. (2013)	1		3		1	80
Straub et al. (2014)	2		1		1	0.36
Tulli et al. (2013)	1		1			1600
Wang et al. (2011)	2		2			100
Yang et al. (2012)	6		3			3300
Yao et al. (2012)	2		8			110
Zhou et al. (2013a)	2		1			67
Zhou et al. (2013b)	1		2			80
Responses	63	7	97	4	4	–
Studies	26	7	26	3	4	–

### Predictor variables

We defined several categorical variables and one continuous variable.

1. Trophic level, which indicated whether the arthropod was a predator or a herbivore (categorical variable).
2. Specialization, which indicated whether the arthropod was a specialist or a generalist (categorical variable).
3. Arthropod response type: the arthropod response type included abundance or diversity for the predators or herbivores and plant damage for herbivores (categorical variable).
4. Habitat: the type of agroecosystem included natural habitats, noncrop habitats, and crop habitats (categorical variable).
5. The scale, within the range of local scales, at which processes were studied (continuous variable).

### Analysis

The degrees of freedom (df), *P*‐value (*P*), *t*‐value (*t*), or coefficient of determination (*r* ^2^) from each response reported in a study were converted into a standard statistic, the correlation coefficient *r*. Then, we computed Fisher's *Z*, using the equation of Rosenthal and DiMatteo (2002): *Z* = 1/2 log[(1 + *r*)/(1–*r*)]. *Z*, the effect size, estimates the magnitude of the relationship between a predictor variable and its response. In this search, we generated 175 effect sizes (*Z*) from 32 studies. We analyzed the relationship between each effect size and the variable responses using Generalized Linear Models (GLM). Statistical analyses were performed with R 2.15.0 (R Development Core Team 2014) and with an alpha level of 0.05.

## Results

### Predator and herbivore response to plant diversity

**Figure 1:**
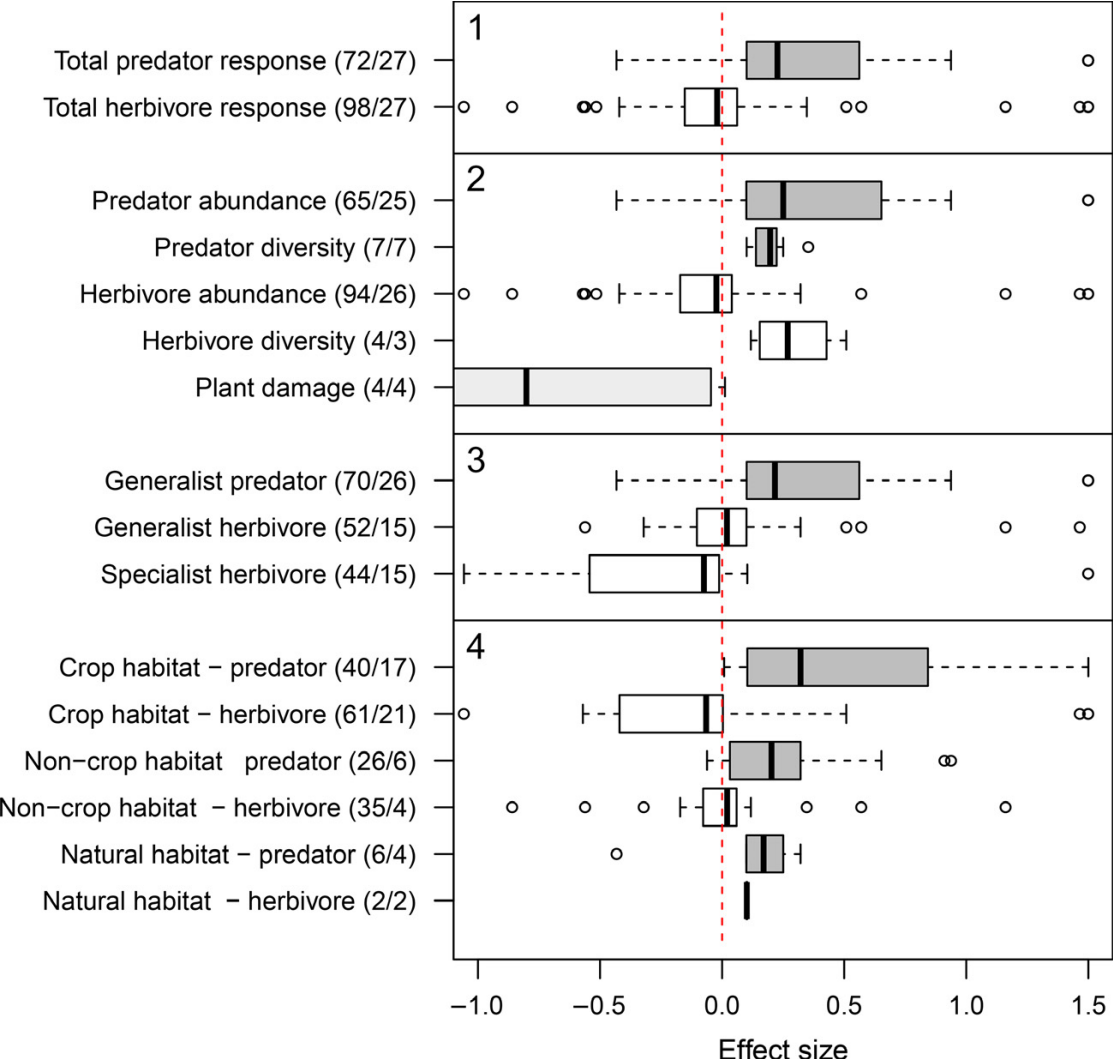
Arthropod responses to plant diversity based on 32 studies and 175 total responses. Numbers in parentheses indicate total number of responses/total number of studies, respectively. The predators, herbivores, and plant damage are in gray, white, and light gray, respectively. Square 1 presents the total predator and herbivore responses (i.e., both abundance and diversity). Square 2 presents the responses of diversity and abundance of predators and herbivores. Square 3 presents the responses of generalist and specialist herbivores. Square 4 presents the responses of predators and herbivores according to the type of habitat (crop, noncrop, natural).

The predators responded positively to increases in plant diversity (*P* = 0.0005, *t* = 3.567, df = 171), while the herbivores responded negatively to increases in plant diversity (*P* = 0.0477, *t* = 1.994, df = 171). Both predator abundance (*P* = 0.00035, *t* = 3.649, df = 169) and predator diversity (*P* = 0.0245, *t* = 2.27, df = 169) responded positively to increases in plant diversity. Herbivore abundance did not have a significant response to plant diversity, but the trend of the response was negative, while herbivore diversity responded positively to increases in plant diversity (*P* = 0.0285, *t* = 2.209, df = 169). Plant damage responded negatively to increases in plant diversity (*P* = 0.0033, *t* = −2.98, df = 171) (Fig. 1). The response of predators and herbivores to plant diversity significantly differed according to trophic level and arthropod response type (Table 2, models 1 and 2, respectively).

**Table 2:** Models tested to determine the effects of predictor variables on the effect size, that is, on the response of predators and herbivores to plant density

Model	Predictor variable	Numbers of papers, observations	df	Residual deviance	*P*‐value (>|*F*|)
1	Trophic level	32, 172	2	79.59	<0.00001
2	Arthropod response type	32, 173	2	94.18	0.04147
3	Specialization	32, 173	2	87.967	<0.00001
4	Scale	32, 162	1	94.839	0.02309
5	Trophic level × arthropod response type	32, 170	1	78.407	0.1138
6	Trophic level × Specialization	32, 169	1	78.493	0.9359
7	Arthropod response type × specialization	32, 169	1	86.353	0.3753
8	Trophic level × scale	32, 169	2	78.631	0.4841
9	Arthropod response type × scale	32, 170	2	91.495	0.80591
10	Specialization × scale	32, 170	2	81.694	0.007953
11	Trophic level × arthropod response type × specialization	32, 165	1	76.980	0.6587
12	Trophic level × arthropod response type × scale	32, 165	1	77.343	0.8429

### Effect of Specialization of arthropods on their responses to plant diversity

The specialization showed a significant response to plant diversity (Table 2, model 3). Generalist arthropods (predator and herbivore considered together) responses to plant diversity were positive (*P* = 0.0102, *t* = 2.597), while it was not significant for specialist arthropods (*P* = 0.4663, *t* = 0.730). A positive response to increases in plant diversity was detected for both generalist predators and herbivores (*P* = 0.0275, *t* = 2.224, df = 168 for generalist predators; *P* = 0.000457, *t* = 3.575, df = 168 for generalist herbivores), but the response was not significant for specialist herbivores (*P* = 0.100, *t* = 1.652, df = 168). The interaction between specialization and the response type (abundance and diversity) was not significantly related to plant diversity (Table 2, model 5). The interaction of specialization with trophic level (Table 2, model 6), arthropod response type (Table 2, model 7), and arthropod response type and trophic level (Table 2, model 11) was not significantly related to plant diversity.

### Effect of scale on arthropod responses to plant diversity

**Figure 2:**
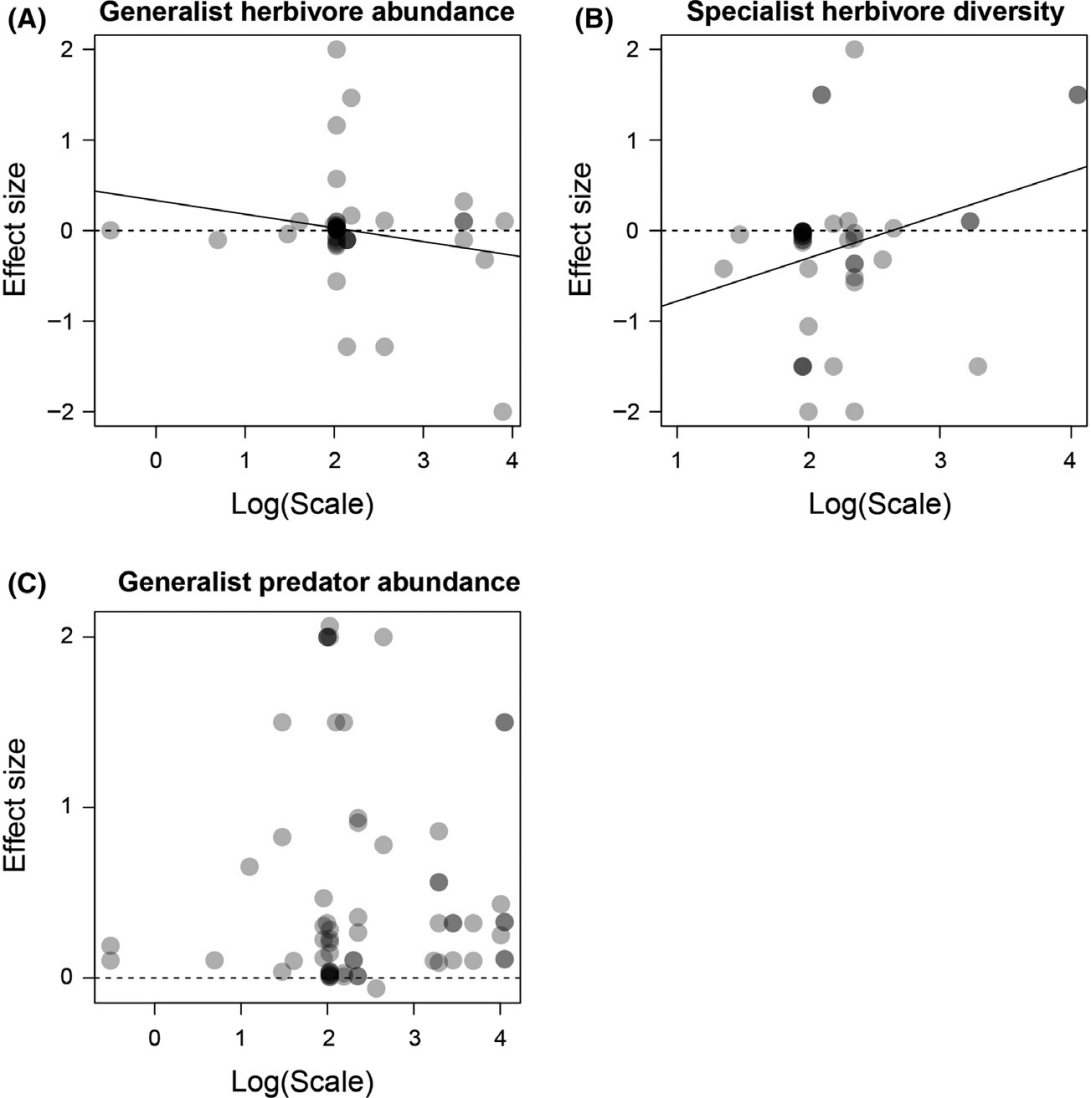
Effect of the scale of observation (log scale in m) on the response of arthropods to plant diversity. (A) specialist herbivore diversity, (B) generalist herbivore abundance, and (C) generalist predator abundance. Solid and broken lines show the linear regression when significant and the zero effect size as reference, respectively. Semitransparency of circles allows representing their eventual superposition.

The response of arthropods to plant diversity was affected by scale (Table 2, model 4) and by the interaction between scale and specialization (Table 2, model 10). The effect size increased with spatial scale for the diversity of specialist herbivores, but decreased with spatial scale for generalist herbivore abundance (Fig. 2A and B). For the abundance of generalist predators, effect size did not change with scale (Fig. 2C). The interactions of scale with trophic level (Table 2, model 8), response type (Table 2, model 9), and trophic level and response type (Table 2, model 12) were not significantly related to arthropod responses to plant diversity.

### Effect of habitat on arthropod responses to plant diversity

Habitat type showed no significant response to plant diversity (*t* = −0.07, *P* = 0.945). Predators did not significantly respond to plant diversity in natural habitats (*P* = 0.193, *t* = −1.13, df = 69), noncrop habitats (*P* = 0.654, *t* = 0.45, df = 69), or crop habitats (*P* = 0.193, *t* = −1.34, df = 69); in all three habitats, however, the trend was positive, that is, predator effect size tended to increase with increases in plant diversity (Fig. 1). Herbivores did not significantly respond to plant diversity in natural habitats (*P* = 0.633, *t* = 0.479, df = 69), noncrop habitats (*P* = 0.900, *t* = −0.127, df = 95), or crop habitats (*P* = 0.845, *t* = 0.196, df = 95); in all three habitats, however, herbivore abundance tended to decrease with increases in plant diversity (Fig. 1).

## Discussion

### Effects of plant diversity on predators and herbivores

**Figure 3:**
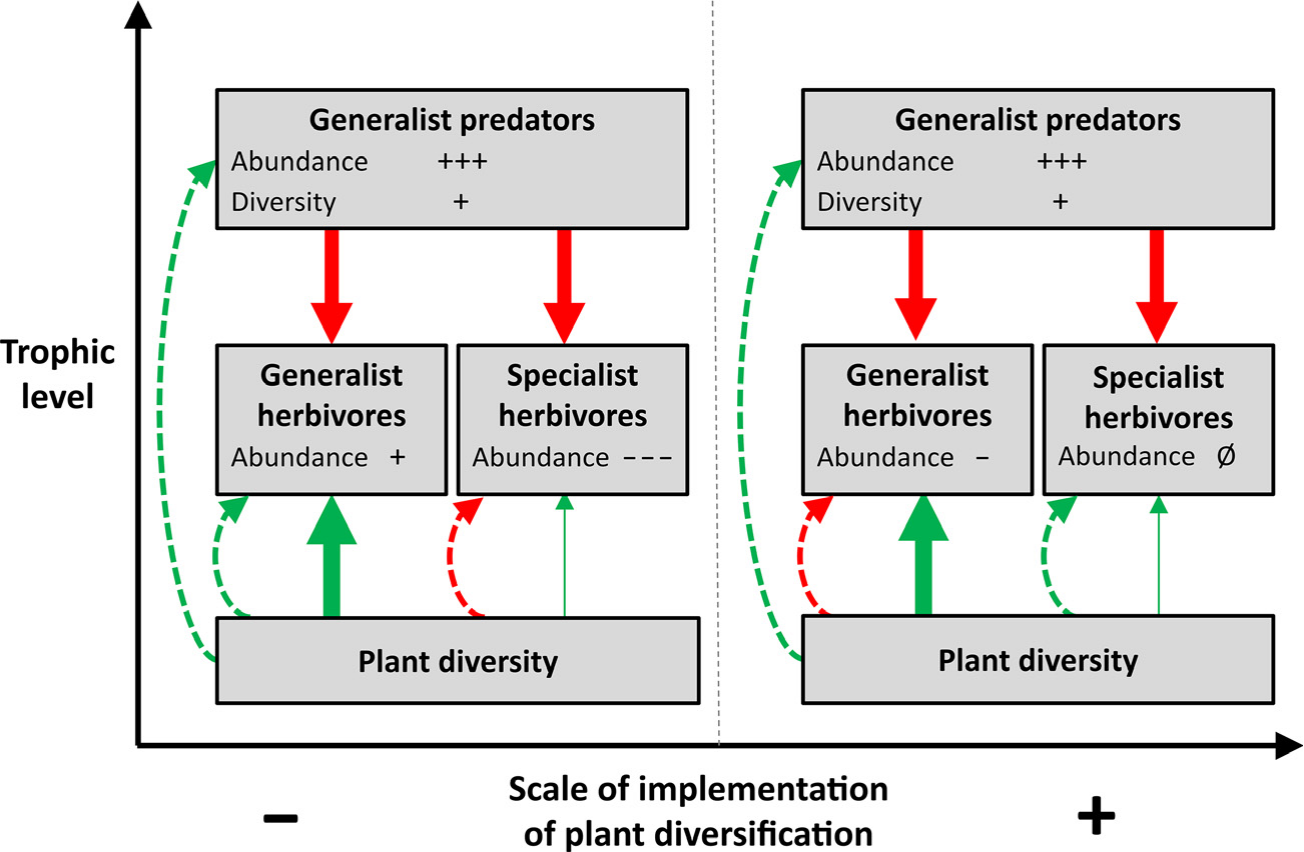
Summary of plant diversity effects according to the trophic level (including the differentiation between generalist and specialist herbivores) and as affected by local scale (from a few square meters to an entire field or field edge). Solid and broken lines show trophic and habitat effects, respectively. Red indicates a positive effect, and green indicates a negative effect. The size of the trophic arrows suggests the strength of the effect. The expected resulting effect on the abundance and the diversity of each trophic group is indicated by signs ranging from “− − −” to “+ + +”.

Our quantitative synthesis of 175 measures of effect sizes reported in 32 papers indicated that plant diversity benefits generalist predators at the local scale. Increases in plant diversity usually cause increases in plant biomass and in habitat diversity, both of which can benefit predators (Hector et al. 1999; Marshall and Moonen 2002). An increase in plant diversity can contribute to an increase in the abundance of predators by providing a broader range and greater abundance of prey (Mollot et al. 2012), of nectar sources, and of suitable microclimates (Landis et al. 2005). Interestingly, there was a contrasted effect of plant diversity on herbivore diversity (positive) and herbivore abundance (neutral). This confirms the positive correlation usually observed between plant diversity and herbivore diversity (Siemann et al. 1998; Hawkins and Porter 2003), while herbivore abundances are more likely correlated with predator abundance and diversity (Letourneau et al. 2011). Our conclusions agree with those of Letourneau et al. (2011), whose meta‐analysis indicated that plant diversity enhances predacious arthropods at scales ranging from the local to the landscape. Like the landscape‐scale meta‐analysis of Chaplin‐Kramer et al. (2011), our meta‐analysis did not reveal a significant response of herbivore abundance to plant diversity. In contrast, herbivore abundance declined with increases in plant diversity in the meta‐analysis of Letourneau et al. (2011). We hypothesize that the absence of an effect of plant diversity on herbivore abundance results from the differing effects of plant diversity on specialist herbivores (the effect was nonsignificant, but tended to be negative) versus generalist herbivores (the effect was significant and positive). This suggests that in the case of generalist herbivores, regulation by generalist predators might be counterbalanced by a strong and positive bottom‐up effect of plant diversity. For specialist herbivores, however, the positive bottom‐up effect in more diversified systems is likely to be smaller and may not counterbalance the increased control by generalist predators. We have summarized these hypotheses in Figure 3. The similarity of our results with the meta‐analysis of Chaplin‐Kramer et al. (2011) supports the hypothesis that the effects of plant diversity on pest control follow similar rules across scales, probably depending on life‐history traits of both predators and herbivores (Tscharntke et al. 2007). Our results highlight the importance of the generalist versus specialist status of herbivores. The distinction between generalist and specialist is also important for predators, that is, specialist rather than generalist predators are better at controlling high densities of herbivores (Diehl et al. 2013), but the opposite is true for low densities of herbivores (Ives et al. 2005).

### Effects of scale and habitat

While the effects of scale remain unclear, the response to plant diversity tended to increase with increases in scale for specialist herbivores, but decrease with increases in scale for generalist herbivores (Fig. 2A and B). More specifically, specialist herbivores tended to respond negatively to increases in plant diversity at smaller scales and positively at larger scales. At larger scales, plant diversification seems to have an overall positive effect on specialist herbivores, which is consistent with the resource concentration hypothesis (Root 1973). At very small scales, the imbrication of plants reduces the resource concentration effect, while at larger scales, the resource concentration effect is dampening the top‐down control by predators. The absence of a significant effect of scale on the response of generalist predators to plant diversity (Fig. 2C) suggests that either (1) the influence of plant diversity occurs at broader scales as shown by Chaplin‐Kramer et al. (2011), or (2) there is no general effect of scale, and the effect of scale is study dependent and probably relies on the dispersal ability of the particular generalist predators in the study site. This absence of a strong response of herbivores and generalist predators to scale strengthens the idea that researchers must assess the most appropriate scale to manage plant diversity for each situation in order to enhance pest control; the appropriate scale is likely to reflect the life‐history traits of the particular predators and herbivores in the specific field. Practical implications of this multiscale management are discussed in Implications for pest control.

Our meta‐analysis did not detect a significant effect of habitat (natural, noncrop, and crop) on the response of predators and herbivores to plant diversity. Plant diversity, however, tended to have greater negative effects on herbivores in crop habitats than in noncrop habitats (Fig. 1). This may be explained by the higher initial plant diversity in noncrop habitats, which would limit the effects of additional diversification. As suggested by Halaj and Wise (2001), trophic cascades are more likely to occur in simplified (agricultural) ecosystems than in natural ones.

### Implications for pest control

Our results confirm that increasing intrafield plant diversity can enhance the control of specialist herbivores by generalist predators. In focusing on maximizing yields, conventional agricultural has depended on low‐diversity cropping systems and on substantial inputs of fertilizer and pesticide. Given an increasingly unpredictable climate, however, maintaining and stabilizing agricultural production are likely to require plant diversification (Isbell 2015). The plant diversification of agricultural fields may be achieved through different means according to the type of cultivated crop (e.g., annual vs. perennial) and the scale of implementation. In the design of plant‐diversified agricultural systems, attention should be paid to the provision of multiple ecosystem services (Gaba et al. 2015) and the need for a social–ecological approach (Lescourret et al. 2015).

Although weed–arthropod interactions have been largely ignored (Bàrberi et al. 2010), a reduced management of weeds (i.e., a reduced dependence on herbicides) is probably the simplest way to increase plant diversity in agricultural systems at the local scale (in a field and along field edges). In the case of perennial crops, intercropping and cover crops are other practices that increase plant diversity and spatial structure of the habitat. Such heterogeneous habitats provide refuge for predators from intraguild predation, thus enhancing pest suppression (Finke and Denno 2006). Cover cropping is a credible option to increase pest control in most temperate and tropical perennial systems, for example, orchards (Aguilar‐Fenollosa and Jacas 2013; Paredes et al. 2015), vineyards (Irvin et al. 2014), and banana plantations (Mollot et al. 2012). In the case of herbaceous annual crops, most options involve the integration field margins and the management of hedgerows (Marshall and Moonen 2002; Gaba et al. 2015). A recent study confirmed the positive effect of grass strips on natural enemies (Sarthou et al. 2014). The integration of trees can greatly increase habitat heterogeneity (Tscharntke et al. 2011). In all cases, however, care must be taken to avoid the integration of plants that result in substantial competition for water and nutrients or in other disservices (Ripoche et al. 2012).

One interesting result of our meta‐analysis was that the response of general predator abundance to plant density was not significantly affected by differences in local scale (Fig. 2C). Along with similar results obtained at higher scales (Chaplin‐Kramer et al. 2011), this suggests that the management of plant diversity at local scales should be guided by technical constraints and the dispersal abilities of the pests and their natural enemies rather than on consideration of a specific scale. It also suggests the need for a multiscale approach, in which temporal and spatial diversification is planned at all scales (Duru et al. 2015). The biodiversification of agroecosystems with the goal of achieving sustainable pest management, as proposed by Lewis et al. (1997), may also be supported by the use of spatially structured, multitrophic, and multiscale models (Tixier et al. 2013). One interesting conclusion of our work also lies in the fact that there are a relatively low number of studies that tend to unravel the role of plant diversity on herbivore control by generalist predators, while it is a major issue in agroecology. This gap of knowledge should be addressed in the future to build the knowledge needed in most agricultural contexts to support the design of more sustainable cropping systems.

In summary, our meta‐analysis confirms that plant diversity has different effects on pest herbivores and their predators. The effects of plant diversity differed greatly between herbivores and predators and also between generalists and specialists within those trophic groups. Spatial scale seems to have no effect or only a moderate effect on the response of arthropods to plant diversity at the local scale. Overall, our results suggest that the response of herbivores to plant diversification reflects a balance between habitat and trophic effects, which depend on arthropod specialization and habitat type.

## Conflict of Interest

None declared.
